# Supplementary material for: Phytochemistry and Bioactivity of Essential Oil and Methanolic Extracts of Origanum vulgare L. from Central Italy
Source: Plants (Basel). 2025 Aug 9;14(16):2468. doi: 10.3390/plants14162468 (PMC12389479; doi:10.3390/plants14162468)
Supplement: Supplementary file 1 [file plants-14-02468-s001.zip › plants-3780734-supplementary.pdf]

Phytochemistry and Bioactivity of Essential Oil and Methanolic Extracts of *Origanum vulgare* L. from Central Italy

Francesca Fantasma*, Marco Segatto, Mayra Colardo, Francesca Di Matteo, Maria Giovanna Chini, Maria Iorizzi and Gabriella Saviano*

Department of Bioscience and Territory, University of Molise, C.da Fonte Lappone snc, 86090 Pesche (IS), Italy

* Correspondence: saviano@unimol.it (G.S.); fantasma@unimol.it (F.F.)

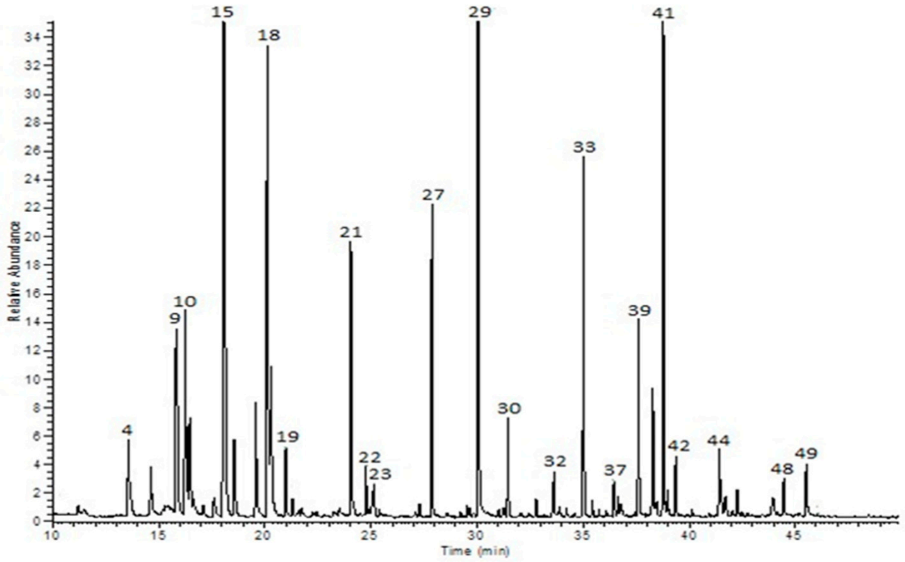

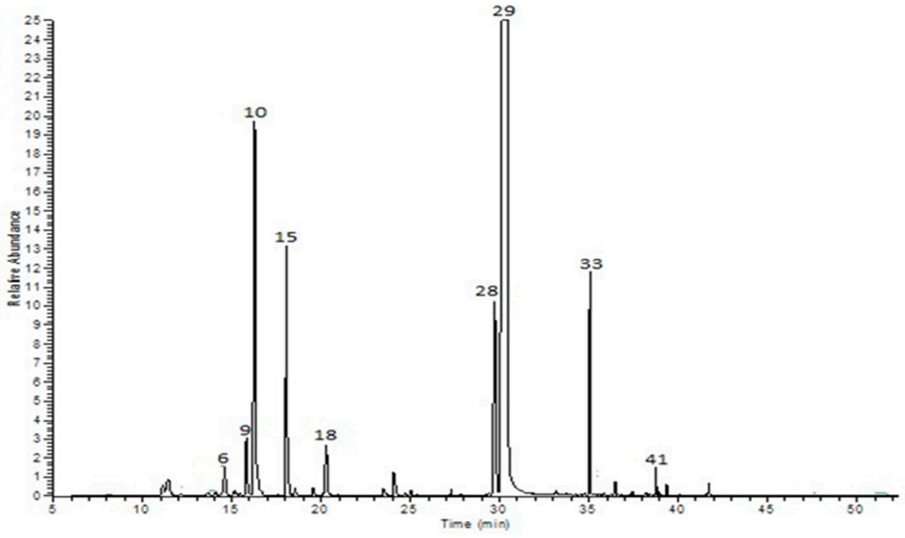

Figure S1: GC-MS TIC chromatogram of wild essential oil (WEO_OR) (top) and commercial essential oil (CEO_OR) (bottom) of the plant species *Origanum vulgare*. The numbers shown on each peak correspond to those in Table 1, allowing the identification of the compounds associated with each peak.

Figure S2 (a-q): HPLC-DAD single chromatograms of 15 phenolic standards.

a) Gallic acid

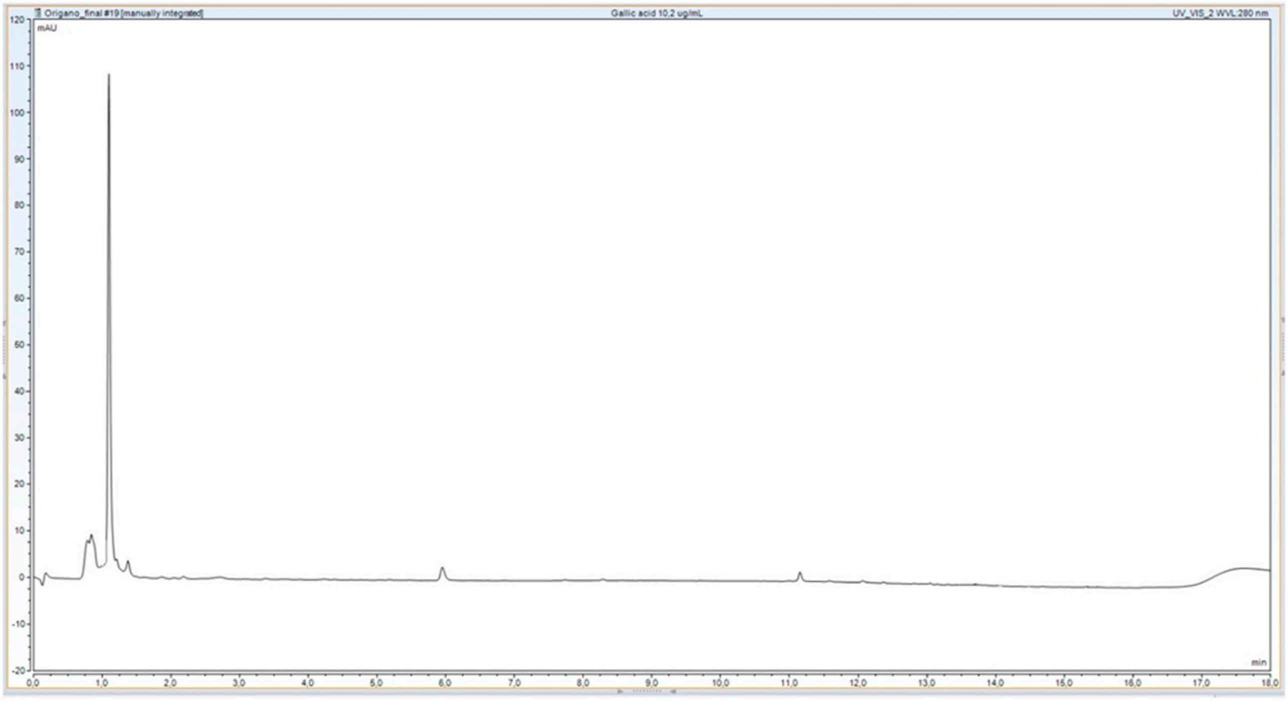

b) Protocatecuic acid

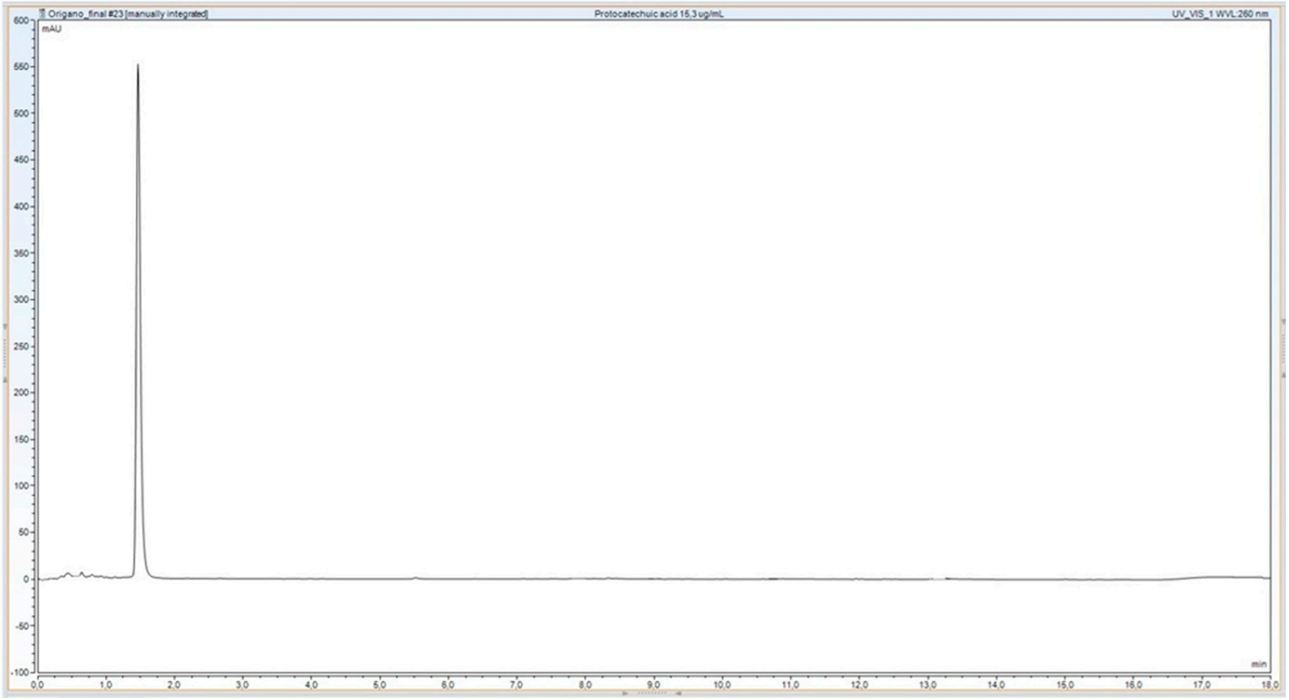

c) 4-Hydroxibenzoic acid

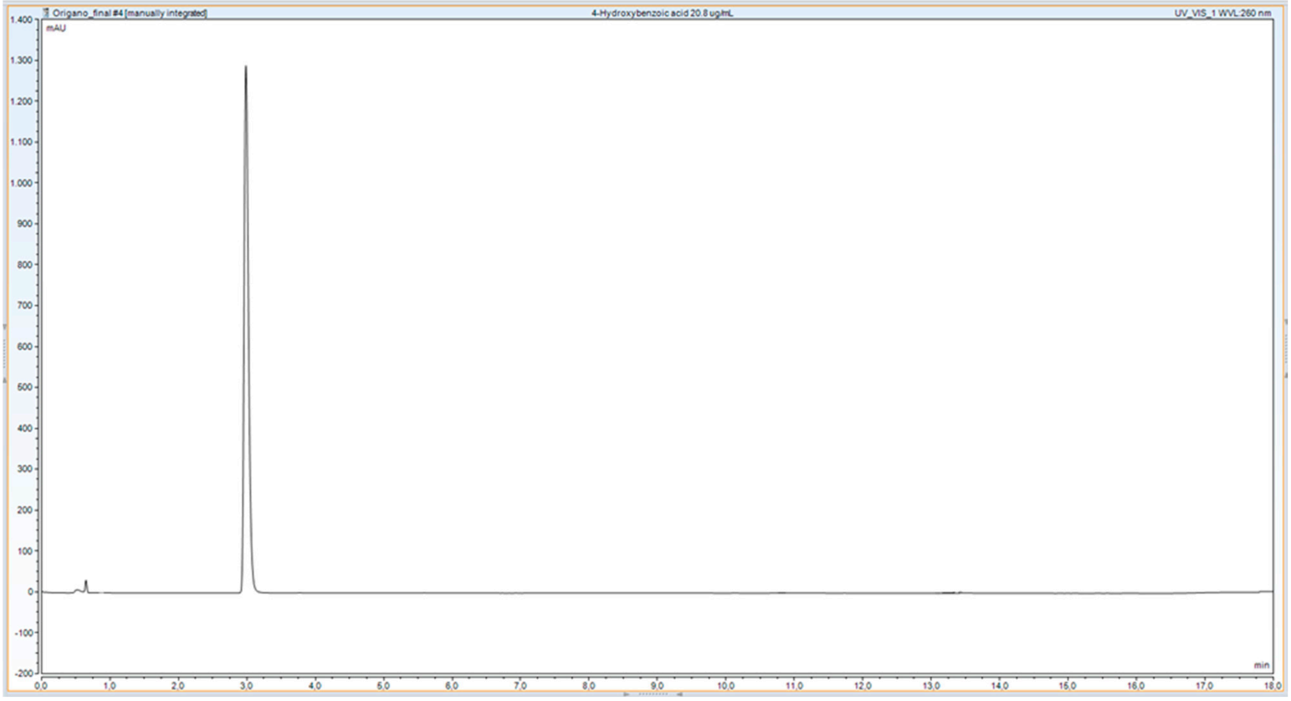

d) Clorogenic acid

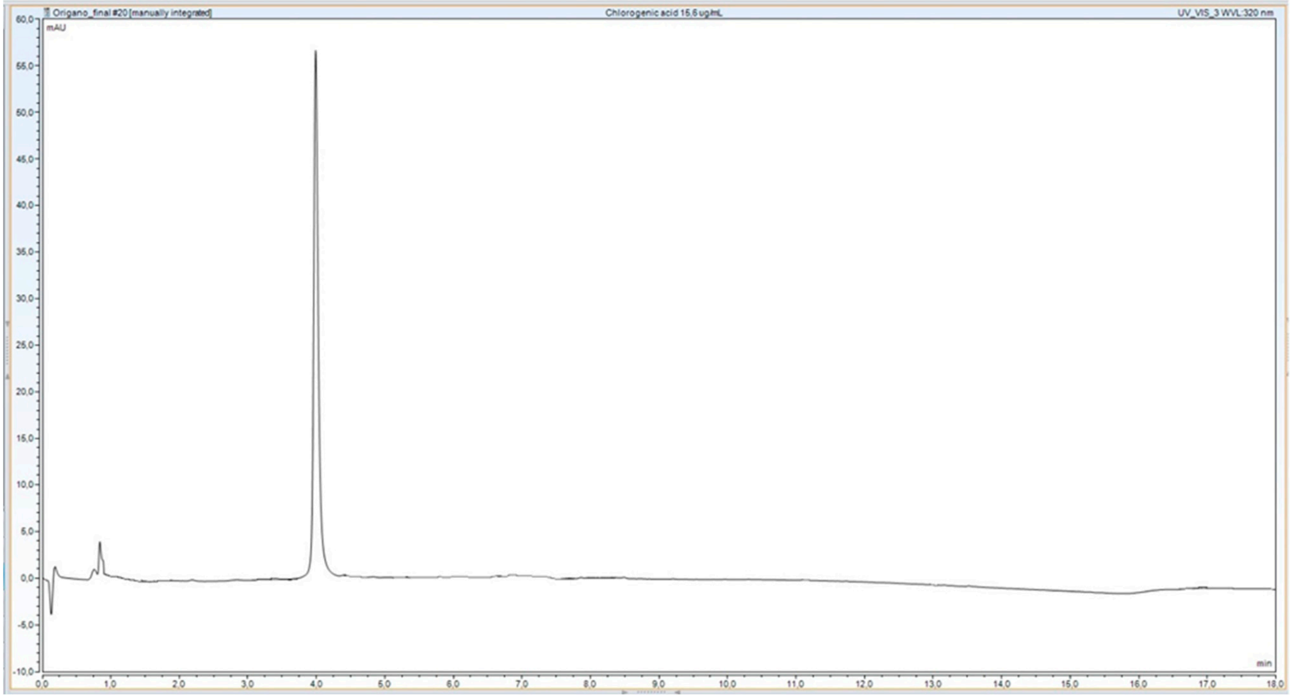

e) Catechin

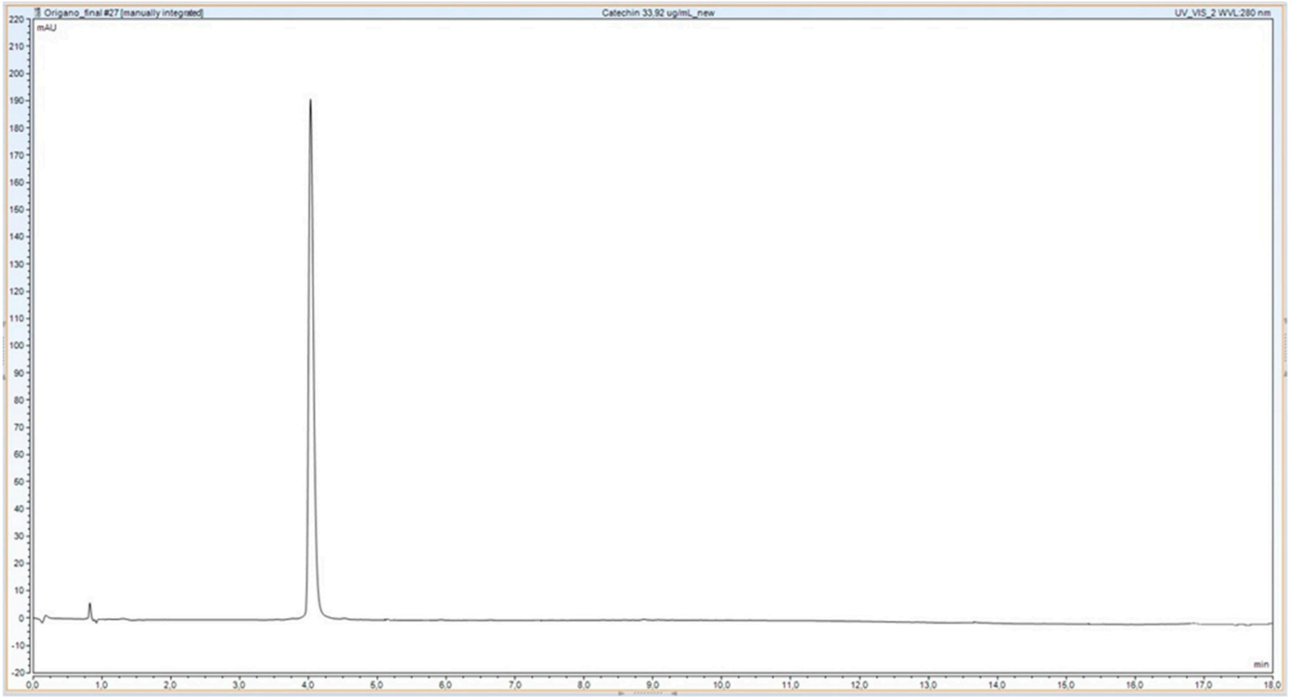

f) Vanillic acid

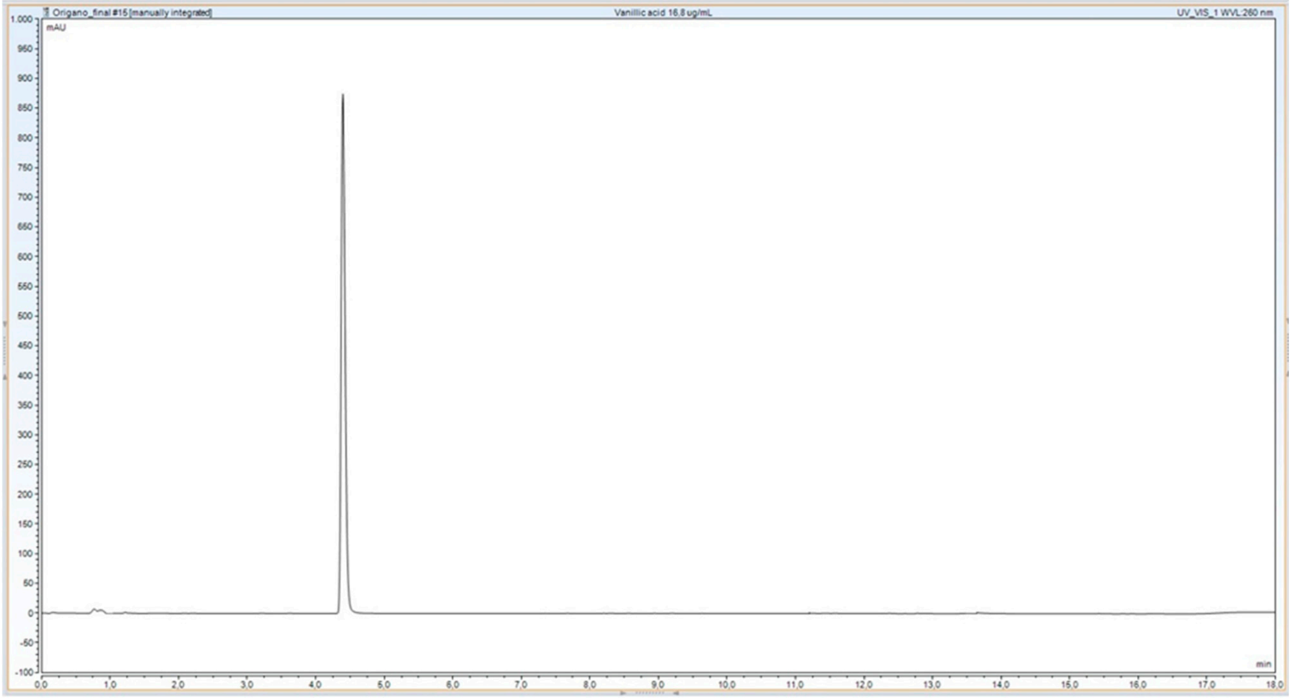

g) Caffeic acid

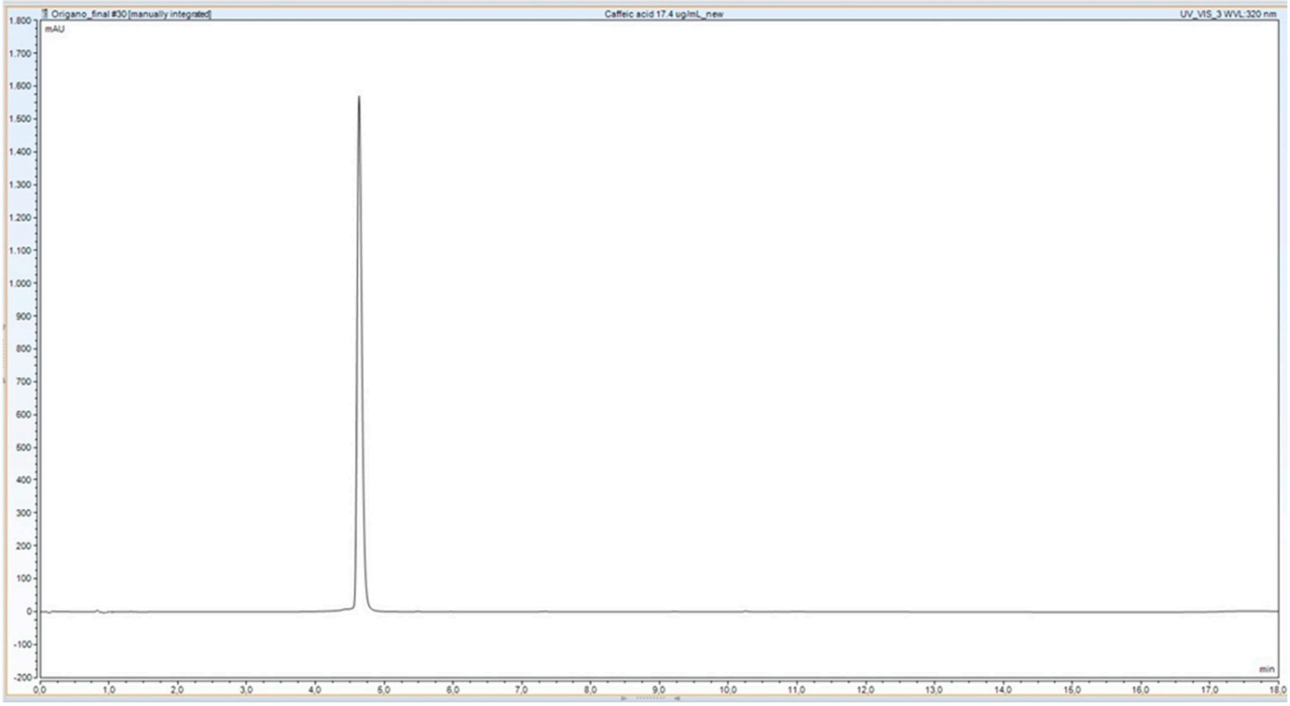

h) Vanillin

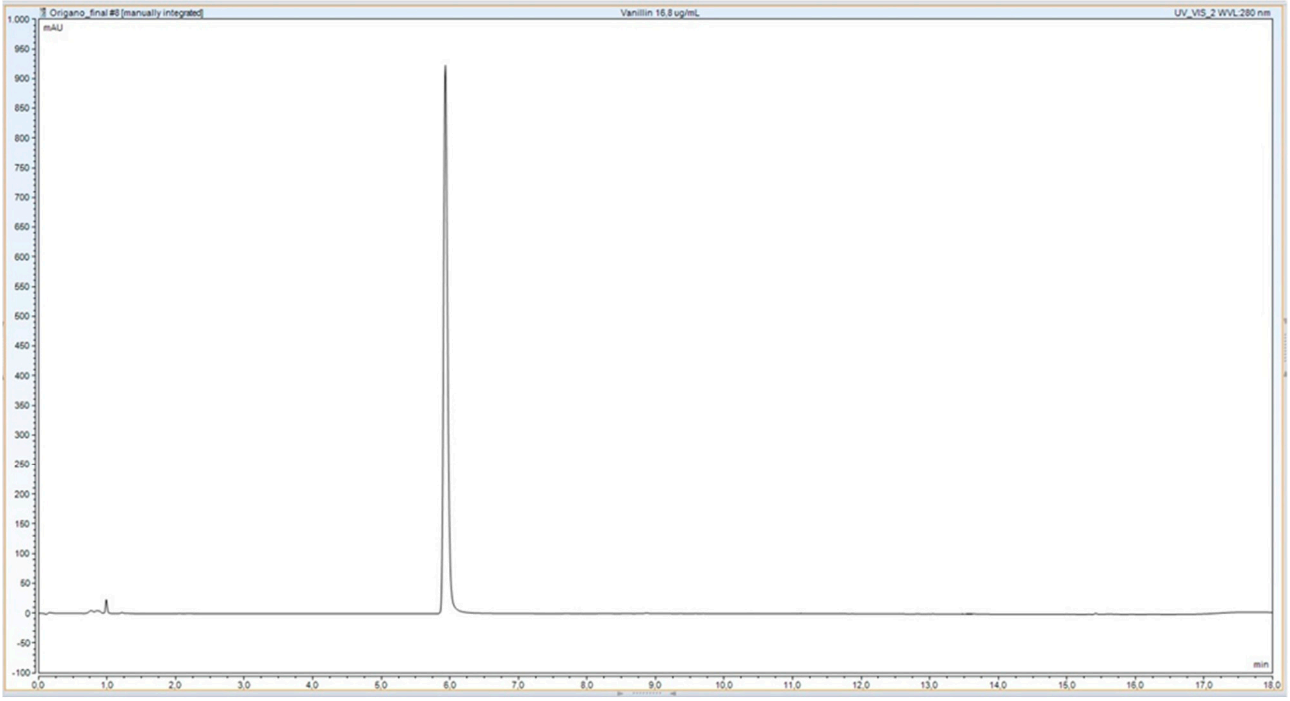

i) *p*-Coumaric acid

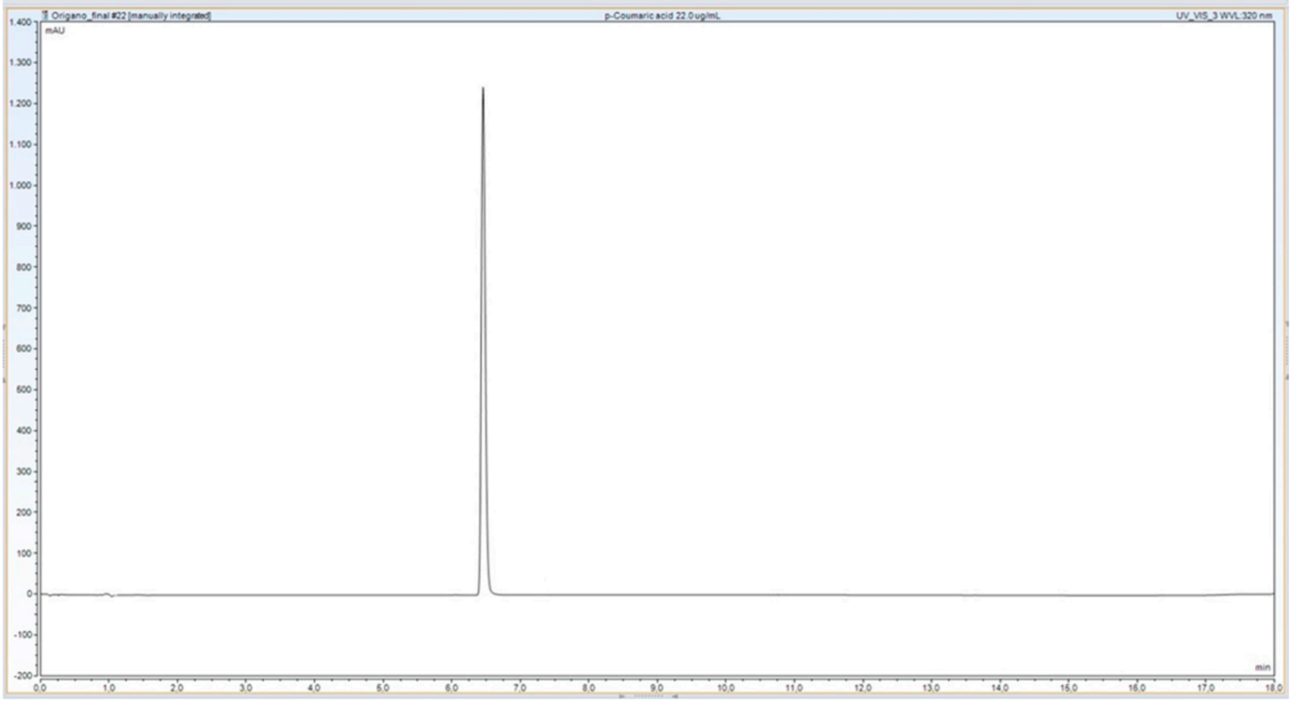

l) Rutin

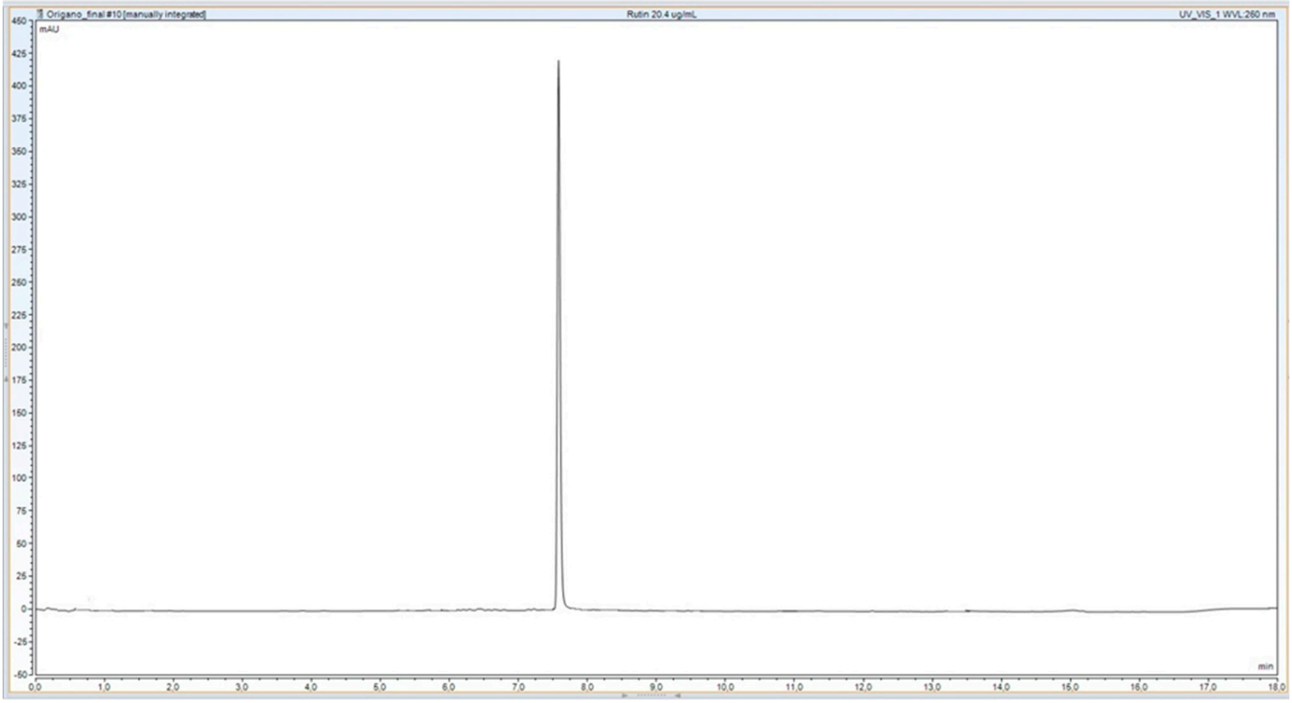

m) Naringin

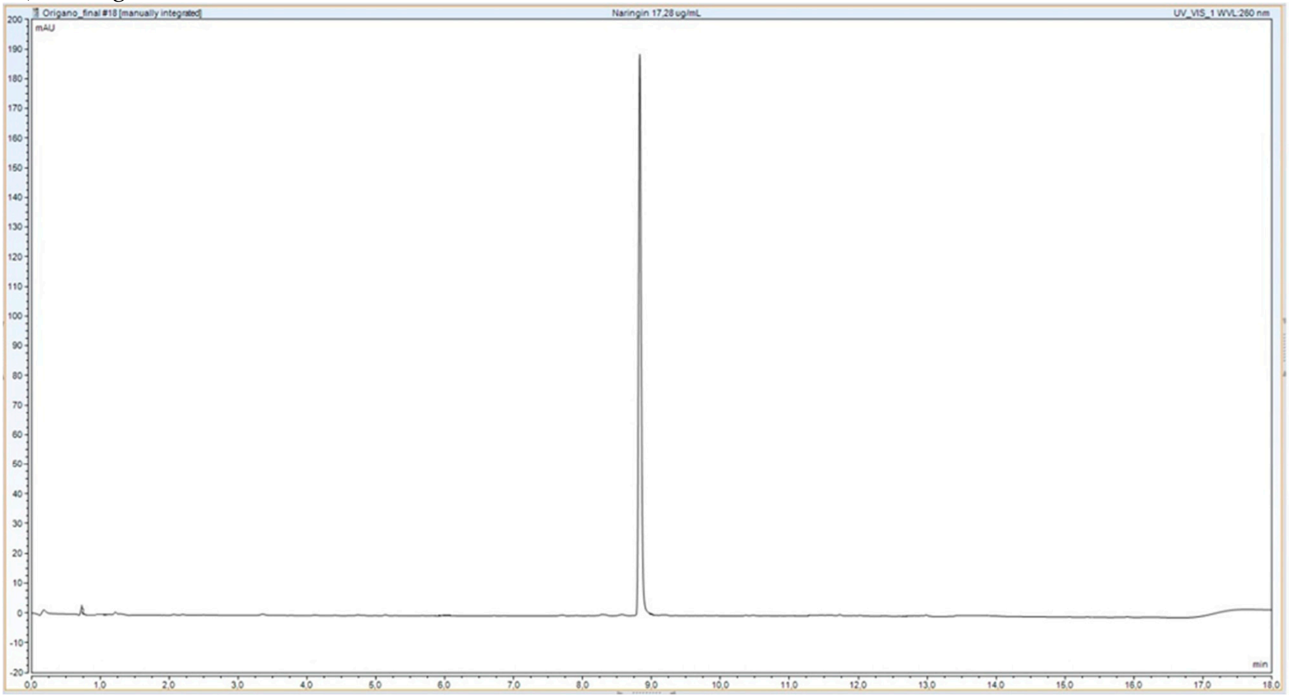

n) Rosmarinic acid

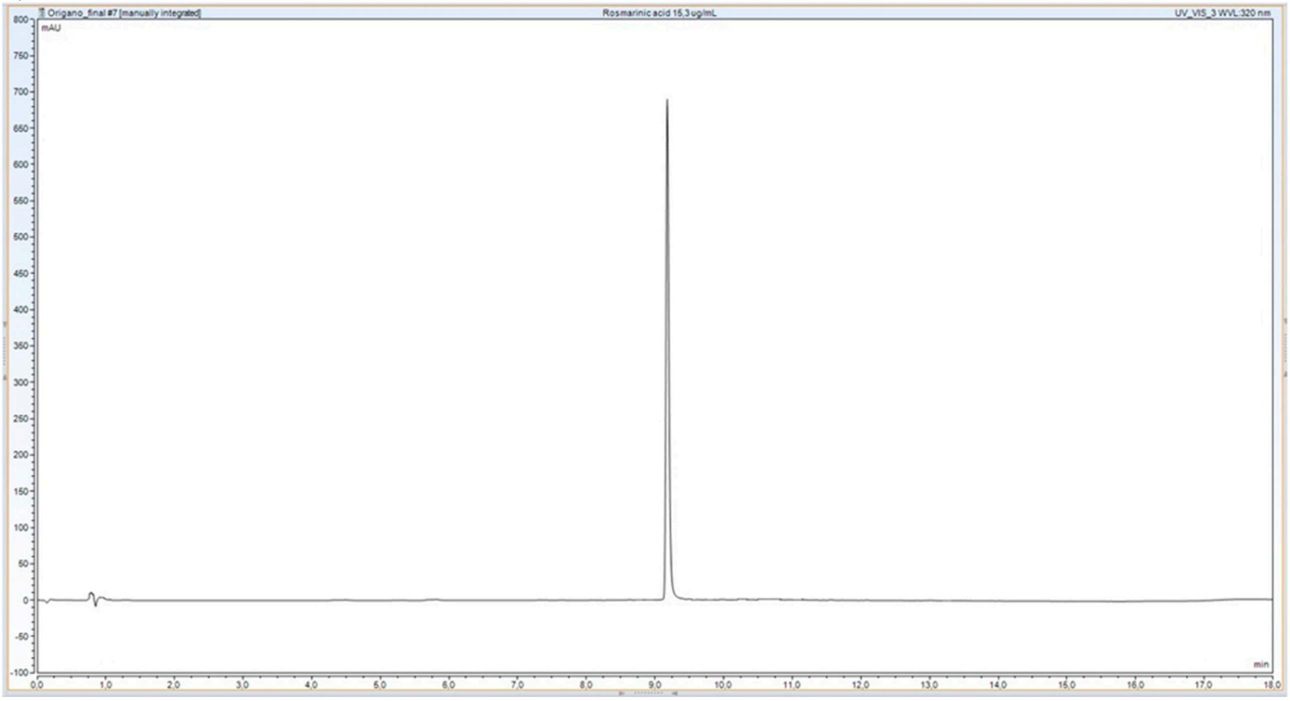

o) Quercetin

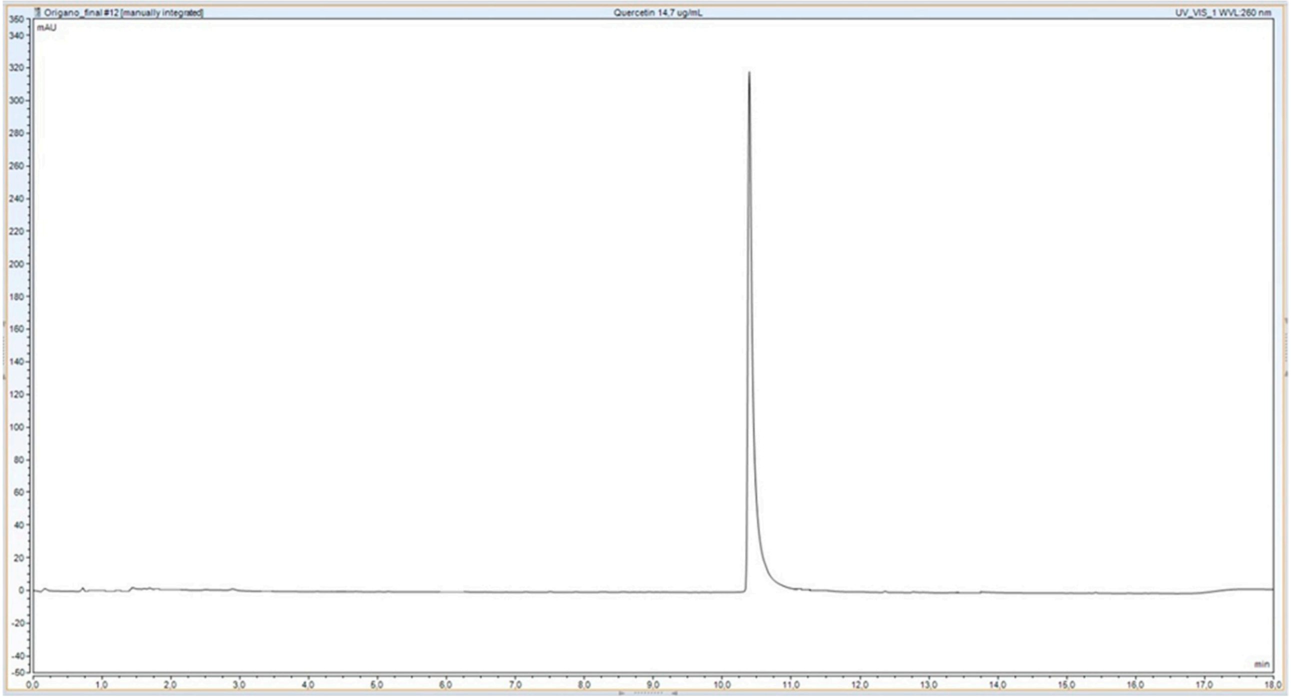

p) Naringenin

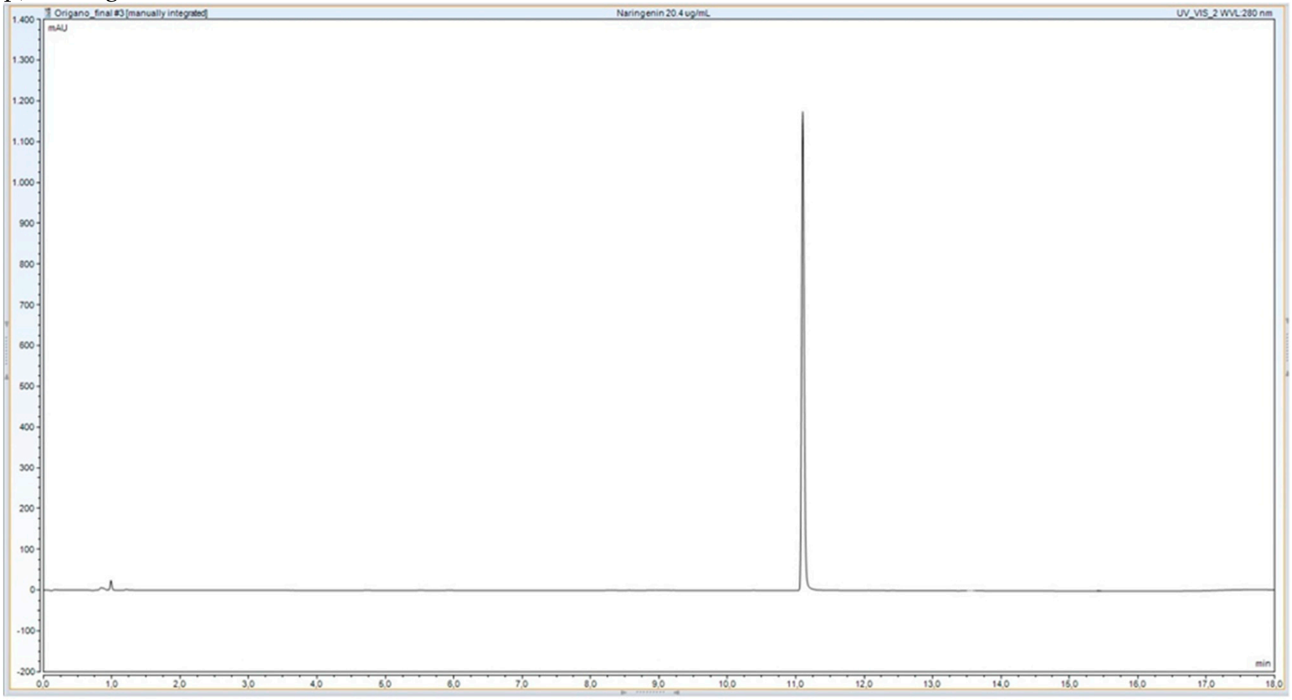

q) Carvacrol

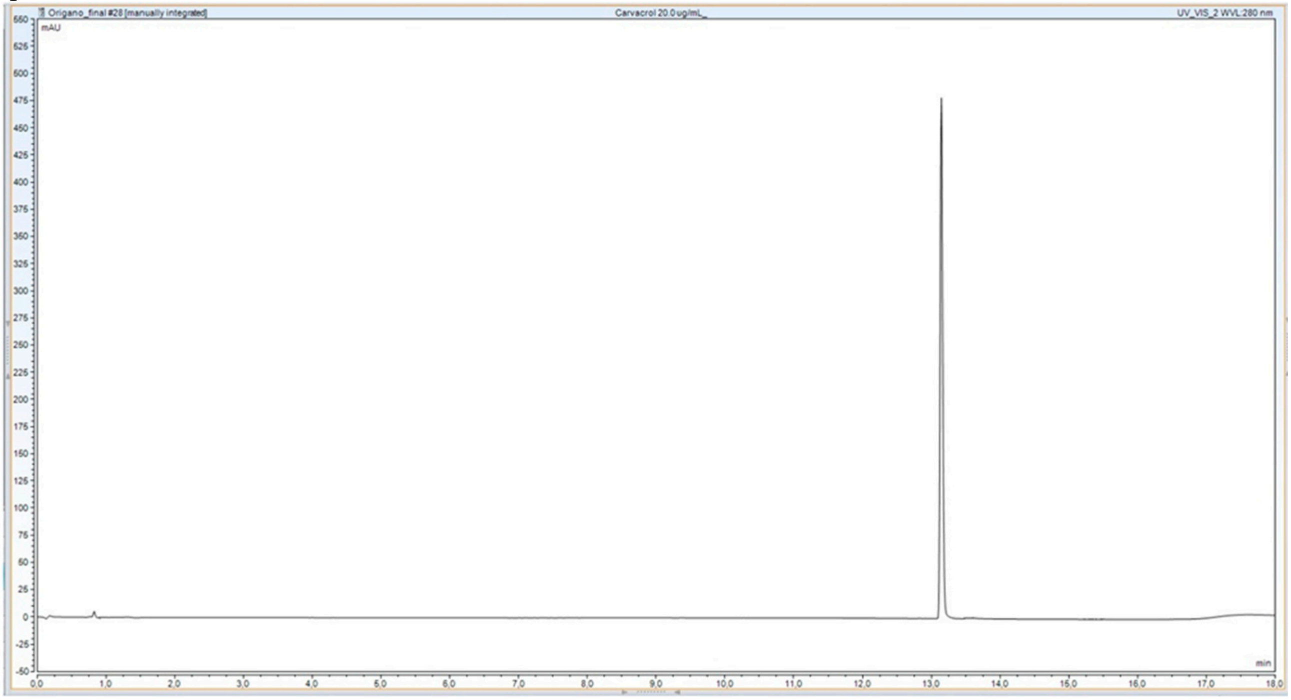
Origano_final #28 [manually integrated]
Carvacrol 20.0ug/mL_
UV_VIS_2 WVL:280 nm
mAU
min

Figure S3: Calibration curves of single 15 standards

GALLIC ACID

Concentrazione (µg/mL)	Area HPLC
5	0,24
13,3	0,625
21,7	1,001
30,0	1,401

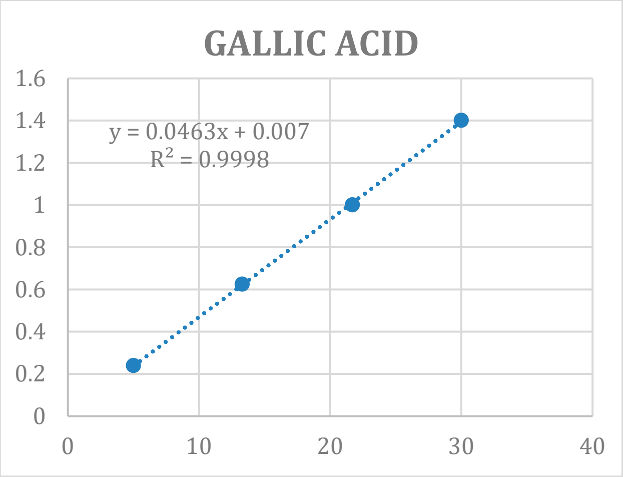

ROSMARINIC ACID

Concentrazione (µg/mL)	Area HPLC
10	0,499
21,7	1,044
33,3	1,631
45,0000	2,108

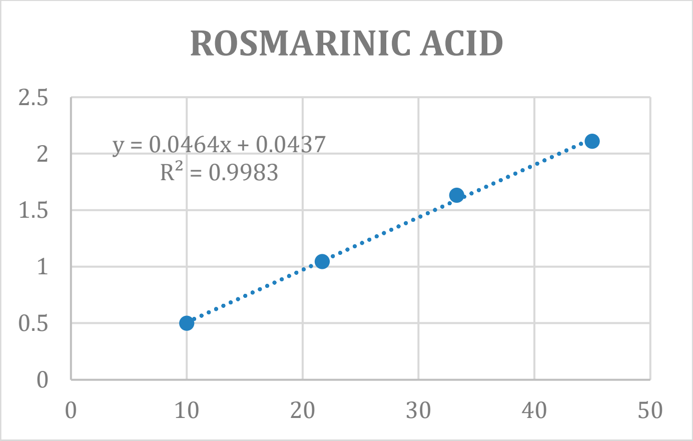

4-HYDROXIBENZOIC ACID

Concentrazione (µg/mL)	Area HPLC
0,2	0,000524
6,8	0,14
13,4	0,269
20,0	0,401

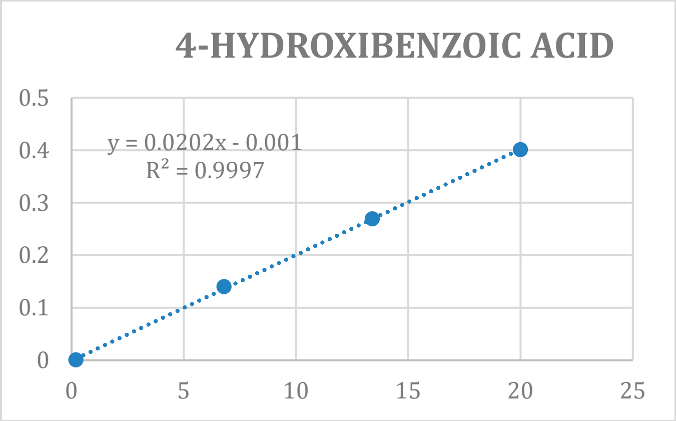

CLOROGENIC ACID

Concentrazione (µg/mL)	Area HPLC
0,2	0,00290
6,8	0,0708
13,4	0,133
20,0	0,202

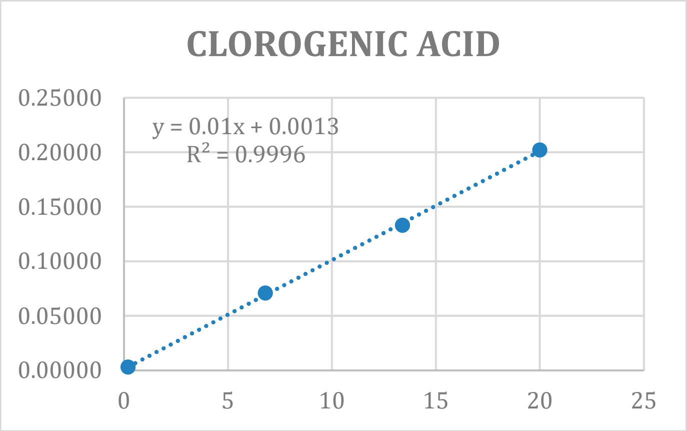

p-COUMARIC ACID

Concentrazione (µg/mL)	Area HPLC
0,2	0,00407
6,8	0,132
13,4	0,266
20,0	0,403

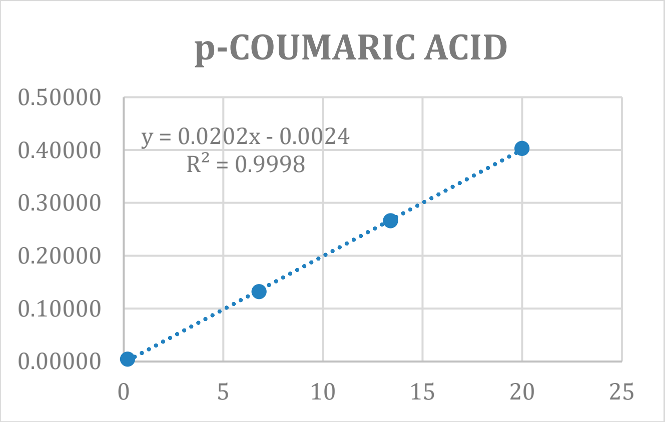

CARVACROL

Concentrazione (µg/mL)	Area HPLC
0,2	0,00414
6,8	0,0993
13,4	0,204
20,0	0,302

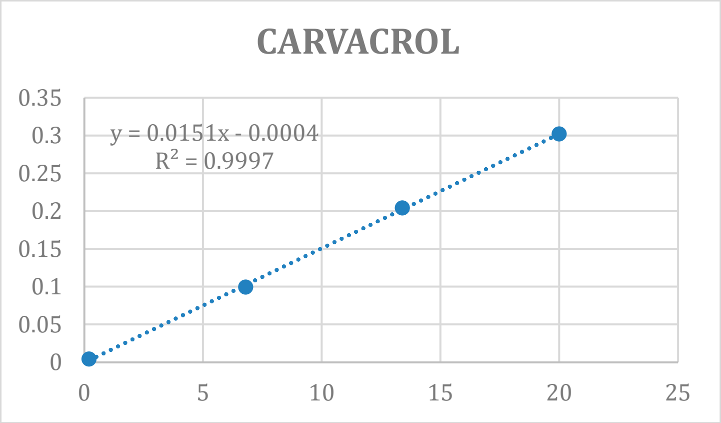

PROTOCATECUIC ACID

Concentrazione (µg/mL)	Area HPLC
0,5	0,0317
7	0,413
13,5	0,783
20,0	1,213

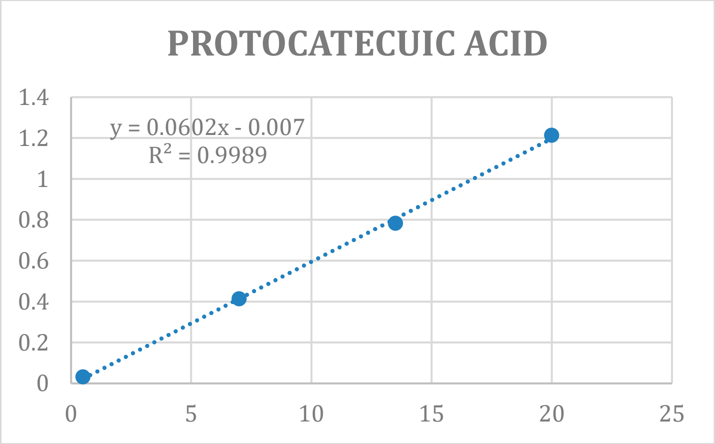

CATECHIN

Concentrazione (µg/mL)	Area HPLC
0,5	0,0452
7	0,807
13,5	1,457
20,0	2,221

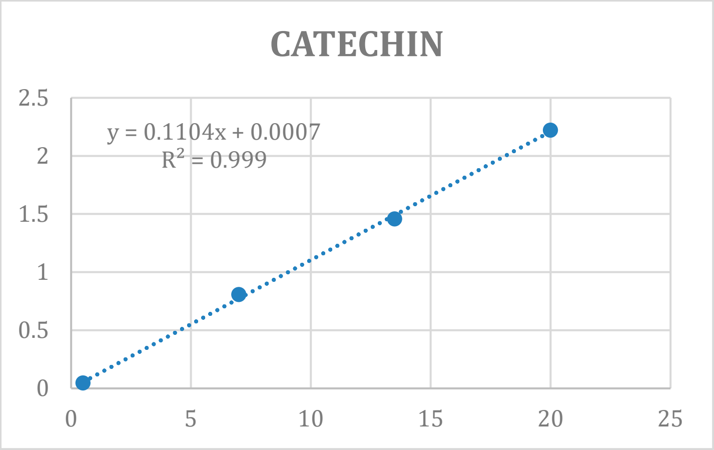

VANILLIC ACID

Concentrazione (µg/mL)	Area HPLC
0,5	0,02500
7	0,32
13,5	0,593
20,0	0,913

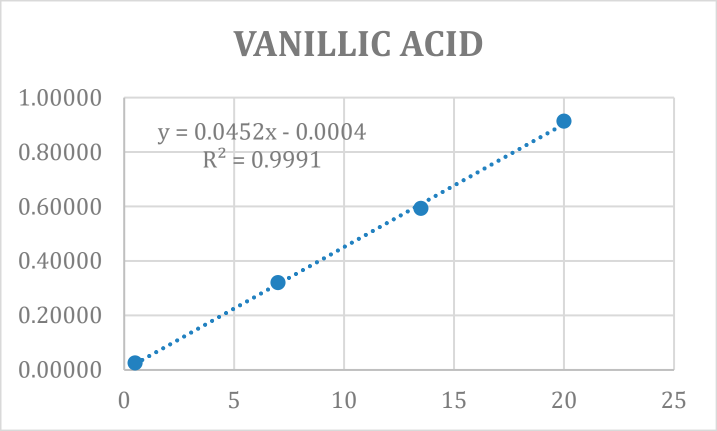

CAFFEIC ACID

Concentrazione (µg/mL)	Area HPLC
0,5	0,01370
7	0,394
13,5	0,713
20,0	1,095

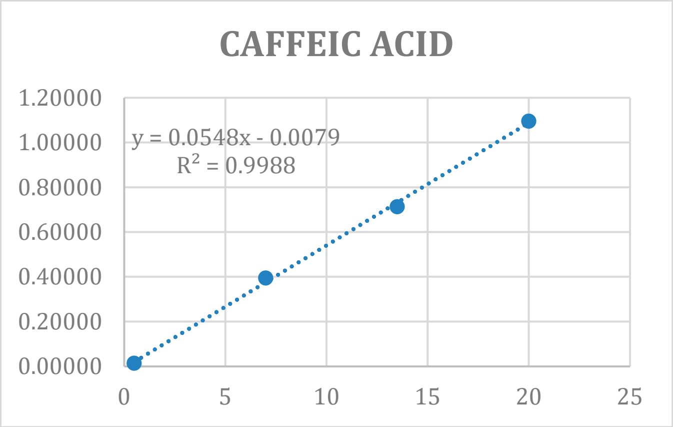

VANILLINE

Concentrazione (µg/mL)	Area HPLC
0,5	0,131
7	2,758
13,5	5,57
20,0	7,939

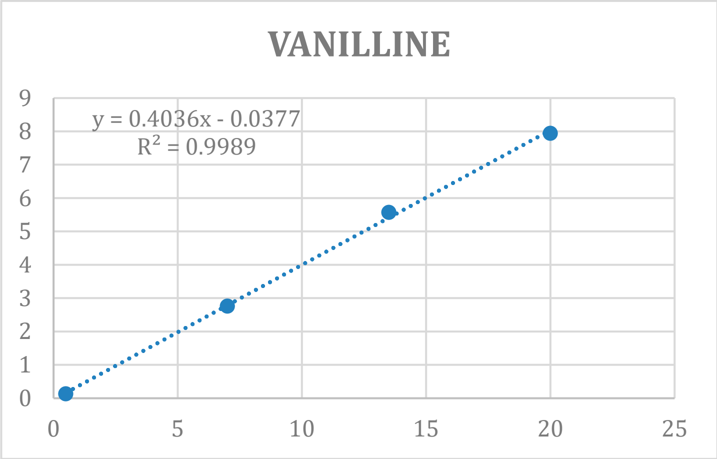

RUTIN

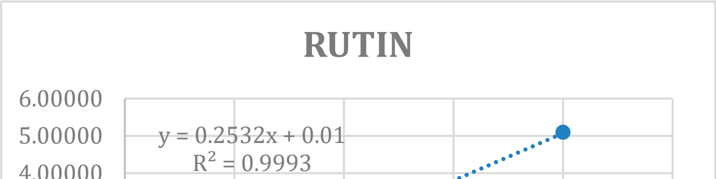

Concentrazione (µg/mL)	Area HPLC
0,5	0,11400
7	1,85
13,5	3,362
20,0	5,097

NARINGIN

Concentrazione (µg/mL)	Area HPLC
0,5	0,14000
7	1,469
13,5	2,886
20,0	4,34

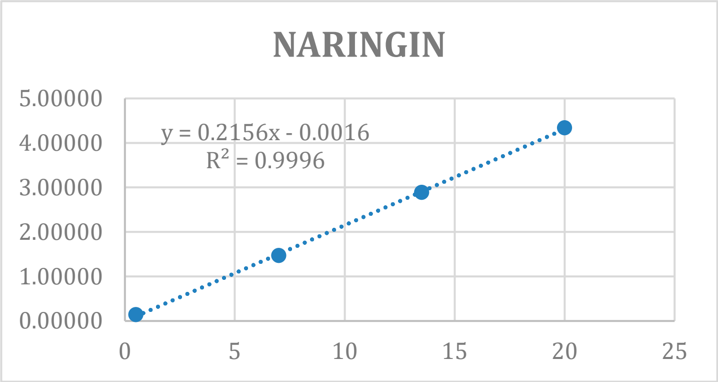

QUERCETIN

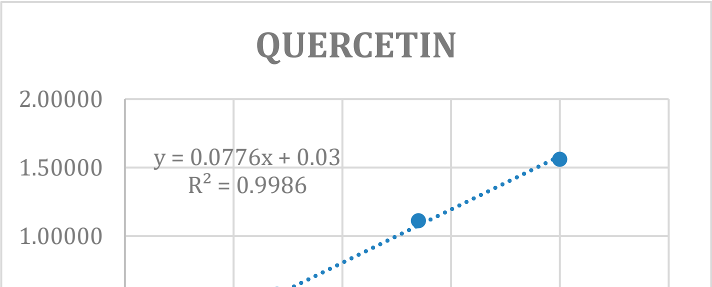

Concentrazione (µg/mL)	Area HPLC
0,5	0,05660
7,0	0,575
13,5	1,112
20,0	1,560

NARINGENIN

Concentrazione (µg/mL)	Area HPLC
0,5	0,04940
7,0	0,394
13,5	0,807
20,0	1,201

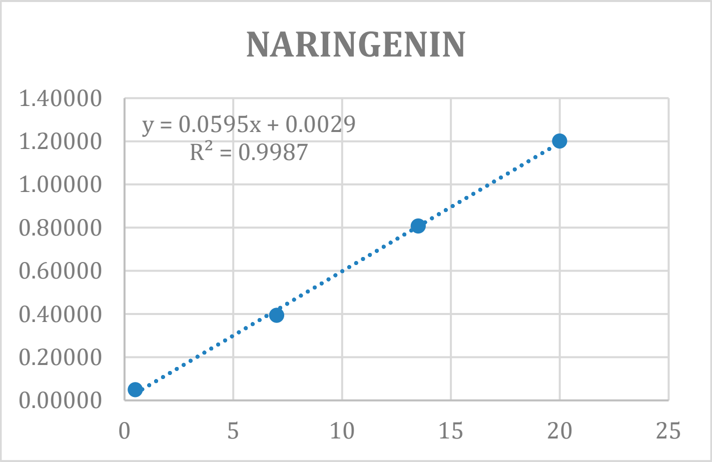

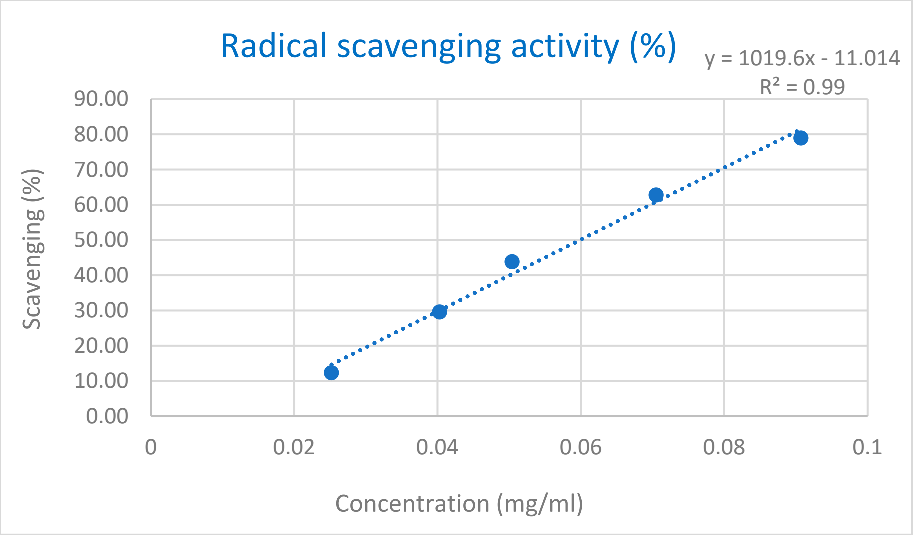

Figure S4. The graph shows one of the three replicates of the methanolic extract obtained for the IC_{50} calculation. The percentage of inhibition as a function of concentration is shown. From the equation of the straight line, knowing the values of m and q, the IC_{50} value can be calculated. IC_{50}= (50- q) /m.

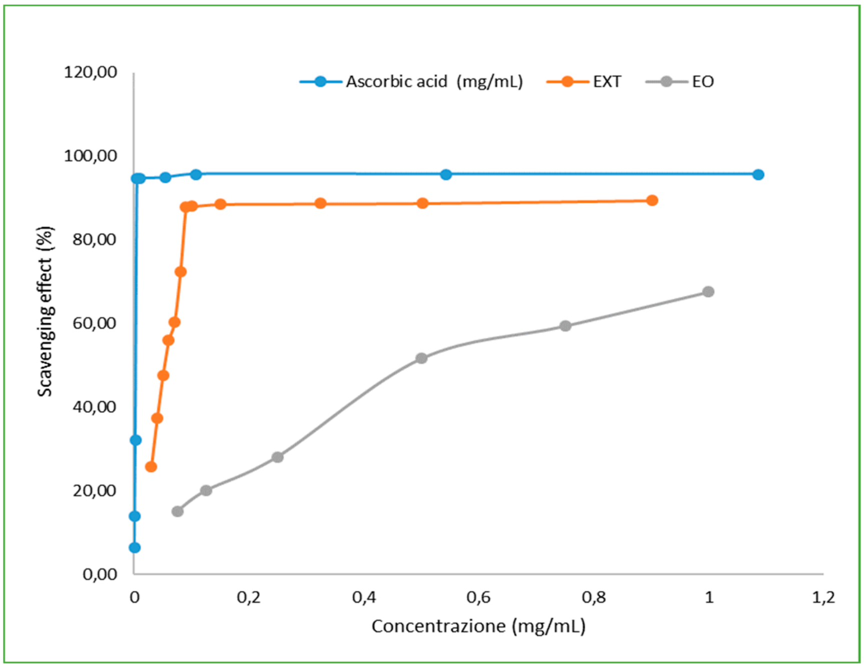

Figure S5. % scavenging of ascorbic acid, methanolic extract of *O. vulgare* and commercial oregano EO, evaluated by the DPPH method.

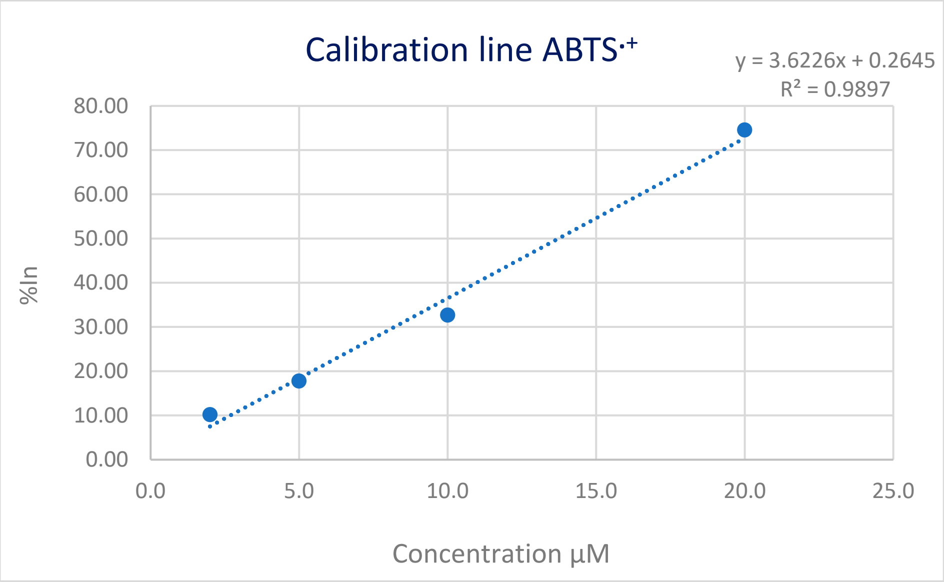

Figure S6. The graph shows the calibration line obtained from the diluted Trolox solutions. m= 3.6226 and q= 0.2645

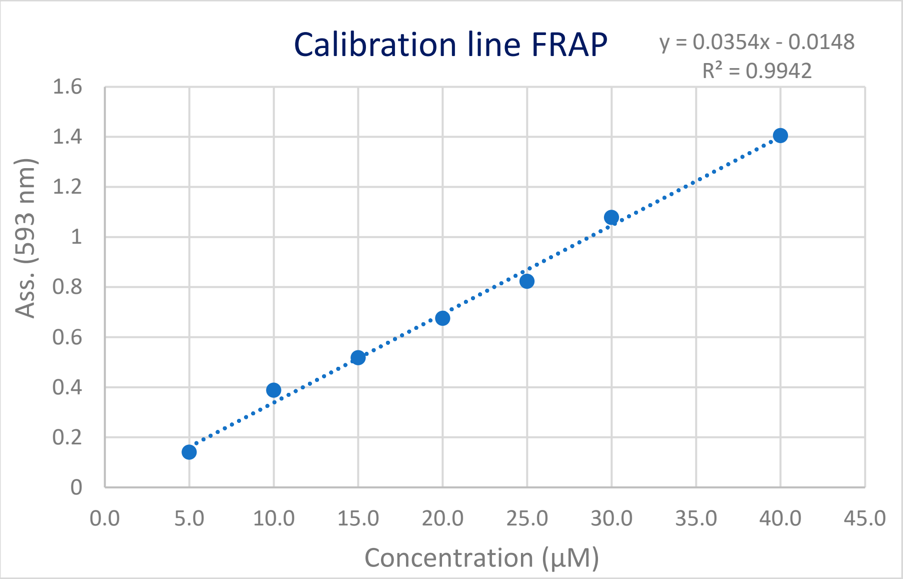

Figure S7. The graph shows the calibration line, constructed from the solutions of diluted Trolox. The value of m= 0.0354, q= -0.0148.

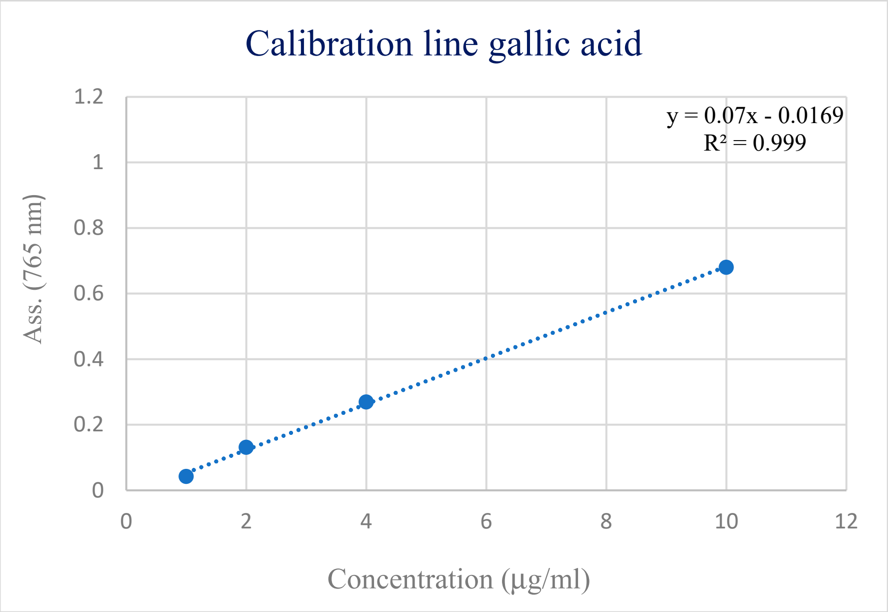

Figure S8. Calibration straight constructed from the dilute solutions of gallic acid. The equation of the straight line allows the values of m= 0.07 and q= -0.0169 to be derived.
